# Knockout of BRD7 results in impaired spermatogenesis and male infertility

**DOI:** 10.1038/srep21776

**Published:** 2016-02-16

**Authors:** Heran Wang, Ran Zhao, Chi Guo, Shihe Jiang, Jing Yang, Yang Xu, Yukun Liu, Liqing Fan, Wei Xiong, Jian Ma, Shuping Peng, Zhaoyang Zeng, Yanhong Zhou, Xiayu Li, Zheng Li, Xiaoling Li, David C. Schmitt, Ming Tan, Guiyuan Li, Ming Zhou

**Affiliations:** 1 Hunan Cancer Hospital and The Affiliated Tumor Hospital of Xiangya Medical School, Central South University, Changsha, Hunan 410013, P.R. China; 2 Cancer Research Institute, Central South University, Key Laboratory of Carcinogenesis, Ministry of Health, Changsha, Hunan, 410078, P.R. China; 3 Institute of reproduction and stem cell engineering, Central South University, Changsha, Hunan, 410078, P.R. China; 4 The Third Xiang-Ya Hospital, Central South University, Changsha, Hunan 410013, P.R. China; 5 Mitchell Cancer Institute, University of South Alabama, Mobile, Alabama 36604, USA

## Abstract

BRD7 was originally identified as a novel bromodomain gene and a potential transcriptional factor. BRD7 was found to be extensively expressed in multiple mouse tissues but was highly expressed in the testis. Furthermore, BRD7 was located in germ cells during multiple stages of spermatogenesis, ranging from the pachytene to the round spermatid stage. Homozygous knockout of BRD7 (BRD7^−/−^) resulted in complete male infertility and spermatogenesis defects, including deformed acrosomal formation, degenerative elongating spermatids and irregular head morphology in postmeiotic germ cells in the seminiferous epithelium, which led to the complete arrest of spermatogenesis at step 13. Moreover, a high ratio of apoptosis was determined by TUNEL analysis, which was supported by high levels of the apoptosis markers annexin V and p53 in knockout testes. Increased expression of the DNA damage maker λH2AX was also found in BRD7^−/−^ mice, whereas DNA damage repair genes were down−regulated. Furthermore, no or lower expression of BRD7 was detected in the testes of azoospermia patients exhibiting spermatogenesis arrest than that in control group. These data demonstrate that BRD7 is involved in male infertility and spermatogenesis in mice, and BRD7 defect might be associated with the occurrence and development of human azoospermia.

Mammalian spermatogenesis is a highly complex process of cell division and differentiation. Spermatogonia undergo several rounds of mitosis followed by meiosis of spermatocytes and spermiogenesis of spermatids in the seminiferous epithelium and subsequent release into the lumen^1^. During spermatogenesis, there are a series of changes associated with the differentiation of haploid round spermatids to spermatozoa. Murine spermiogenesis can be divided into four phases on the context of sharp nuclear condensation: round spermatid (steps 1–8), elongating spermatid (steps 9–11), condensing spermatid (steps 12–13) and condensed spermatid (steps 14–16)^2^. During late spermatogenesis, morphological changes in the nucleus content of spermatids require the involvement of chromatin remodeling factors and histone acetylase^3^,^4^. When nucleosome DNA-containing histones are highly supercoiled, they are replaced first by the transition proteins TP1 and TP2 and subsequently by Prm1 and Prm2^5^.

The number of germ cells in the seminiferous tubules is determined by a dynamic balance between cell proliferation and apoptosis^6^. Apoptosis plays an important role in regulating spermatogenesis of various mammalian species, including humans^7^. Testicular germ cell apoptosis occurs normally and continuously throughout life. In addition, external or internal disturbances such as cryptorchidism, genetic alterations, irradiation or exposure to toxicants, alterations of hormonal support, heat exposure and treatment with chemotherapeutic compounds result in increased germ cell apoptosis^8^,^9^. A dramatic increase in germ cell apoptosis occurs in response to several pathological conditions, including idiopathic infertility in males^10^. Apoptotic markers can potentially be used to assess the fertilization rates of spermatozoa. For example, increased Annexin V and DNA fragmentation (λH2AX) serve as important markers for sperm survival and the ability to fertilize^11^. Accumulating DNA damage initiates apoptosis cascades such as the p53 signaling pathway.

BRD7 was first identified as a novel bromodomain gene with a single bromodomain related to multiple types of cancers^12^,^13^. As a tumor suppressor gene, BRD7 inhibits cell growth and cell cycle progression from G1 to S phase through transcriptional regulation of the Ras/MEK/ERK, Rb/E2F, and Wnt/β-catenin pathways in NPC cells^14^. BRD7 has been identified as a co-factor of p53 and is required for the efficient induction of p53-dependent oncogene-induced senescence in breast cancer^15^,^16^. Furthermore, as a subunit of the SWI/SNF chromatin-remodeling complex, BRD7 can specifically bind to acetylated lysines on the N-terminal tail of histones H3 and H4 to affect the transcription of numerous genes^17^,^18^,^19^. In this study, we generated a BRD7-knockout mouse model by using the *Cre*/*loxP* and *Flp*/*FRT* recombination systems, which we used to study the role of BRD7 in spermatogenesis *in vivo*. We observed multiple defects in post meiotic germ cells, including acrosome deformation, abnormal head shape and degenerative spermatids as well as high apoptosis levels in germ cells in BRD7-knockout mice. Moreover, idiopathic azoospermia was characterized by no or low BRD7 expression. Our studies demonstrate the critical role of BRD7 in spermatogenesis and male fertility, suggesting that BRD7 may be a promising target for the diagnosis and treatment of human idiopathic azoospermia patients.

## Results

### BRD7 expression profile during multiple stages of testis development and spermatogenesis

**Figure 1:**
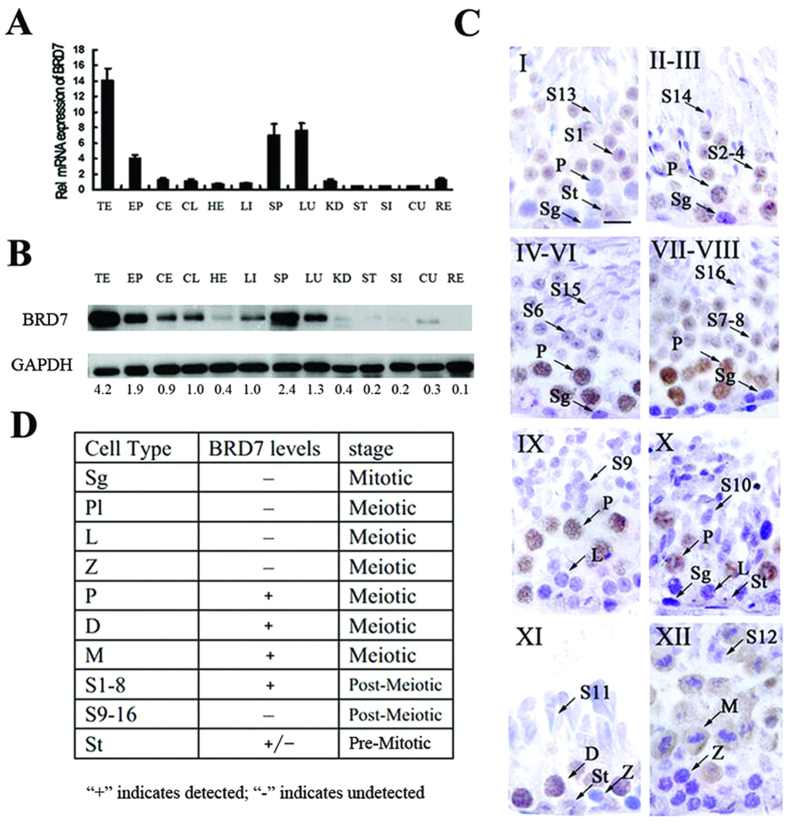
Expression profile of BRD7 during multiple stages of testis development and spermatogenesis. (**A**) Real-time qPCR analysis of BRD7 mRNA expression relative to GAPDH in various tissues from adult C57BL/6 mice. BRD7 expression in the liver was normalized to 1; 3 WT mice were used in the assay. (**B**) BRD7 expression in various tissues detected by western blotting. The quantitative analysis of BRD7 was relative to GAPDH. TE, testis; EP, epididymis; CE, cerebrum; CL, cerebellum; HE, heart; LI, liver; SP, spleen; LU, lung; KI, kidney; ST, stomach; SI, small intestine; CU, cecum; RE, rectum. (**C**) IHC staining of cross sections from seven-week-old wild-type testes with anti-BRD7 antibody. The seminiferous tubules are from Stage I to Stage XII. St, Sertoli; Sg, spermatogonia; PI, Pre-leptotene; L, leptotene spermatocytes; Z, zygotene spermatocyte; P, pachytene spermatocyte; D, diplotene spermatocyte M, meiotic germ cell; S1-8, step 1–8 round spermatid; ES, step 9–16 elongating or elongated spermatids. Scale bar: 20 μm. (**D**) The table shows the immunostaining of BRD7 in different cell types, “+” or “−” indicates BRD7 staining detected and undetected in these cell populations.

BRD7 was detected in adult mouse tissues and primarily expressed in the testis, epididymis, spleen and lung, with extremely high expression levels in the testis (Fig. 1A,B). In adult mouse testes, BRD7 immunostaining was detected in the nuclei of pachytene spermatocytes of stage II to X and diplotene spermatocytes of stage XI, secondary spermatids (stage XII) and round spermatids (steps 1–8). Very low or absent levels of BRD7 immunostaining were also evident in adult Sertoli cell nuclei (Fig. 1C). No BRD7 immunostaining was observed in spermatogonia, leptotene or zygotene spermatocytes of any stages or elongating spermatids (steps 9–16) (Fig. 1C,D). The similar expression pattern of BRD7 was observed among immature seminiferous epithelium (Supplementary Fig. S1A), which was also identified by co-localization of BRD7 andγH2AX (Supplementary Fig. S1B). Western blot analysis showed that the V-shape dynamic expression pattern of BRD7 appeared during postnatal testis development from postnatal days (pn) 7 to 45, and the lowest BRD7 expression level was detected at pn 12, while after pn 14, BRD7 expression increased until pn 23 (Supplementary Fig. S1C). The specific expression pattern of BRD7 in the testis suggests a critical role of BRD7 in mouse testis development and spermatogenesis.

### Generation of the BRD7–knockout mouse model

**Figure 2:**
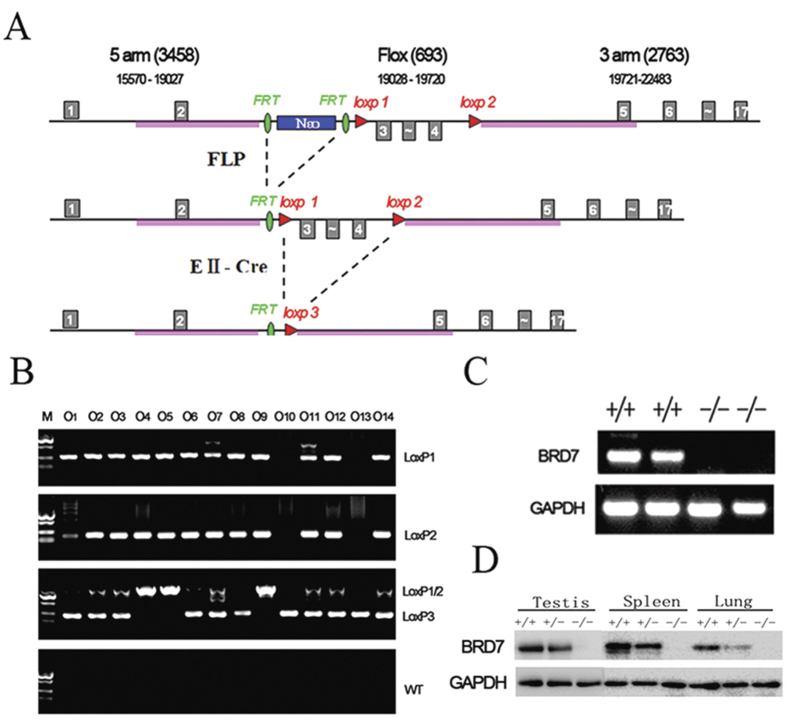
Generation of BRD7-knockout mice and genotype identification. (**A**) Gene targeting strategy (exons are not drawn to scale). Exons 3 and 4 were flanked by *LoxP* sites (red triangles), and a neomycin selection cassette was flanked by *FRT* sites (green oval). (**B**) Genomic identification of WT, Het and KO mice by PCR based on the appearance of different *LoxP* sites. O1 to O14 are 14 representative mice, O10 and O13 are BRD7^−/−^ mice with only the LoxP 3 site. (**C**) Genotype identification of BRD7^+/+^ and BRD7^−/−^ mice by RT-PCR for the 3 and 4 exon sequences. The templates were obtained from the mouse testes. (**D**) Western blot analysis of BRD7 expression in the testis, spleen and lung from BRD7^+/+^, BRD7^+/−^ and BRD7^−/−^ mice.

To obtain BRD7-knockout (KO) mice that allowed for both conditional and global disruption of the BRD7 gene, we used the *Cre*/*loxP* and *flp*/*FRT* recombination systems to target exons 3 and 4 (Fig. 2A). Heterozygous floxed BRD7 mice were verified by PCR and sequence determination (Supplementary Fig. S2A). The exon-3–4-deficient mice were identified by PCR amplification using four primer pairs (Fig. 2B). The primer pairs from exons 3–4 and exon 2 were further used to identify the genotypes by sequencing (Supplementary Fig. S2B) and RT-PCR (Fig. 2C). In BRD7^+/+^ and BRD7^+/−^ testis, spleen and lung extracts, a specific BRD7 band was detected but was absent in the same BRD7^−/−^ mouse tissue extracts (Fig. 2D). This finding was also confirmed by immunohistochemistry (IHC) in the testis (Supplementary Fig. S2C). The above results demonstrate that the BRD7-knockout mouse model was successfully generated.

### BRD7 deficiency leads to male infertility with azoospermia

**Figure 3:**
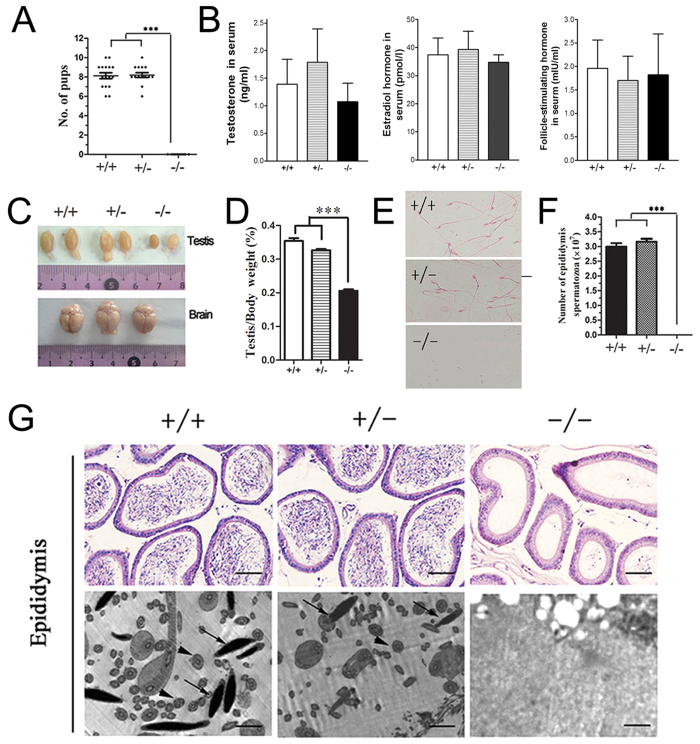
BRD7 deficiency leads to male infertility with a lack of epididymal sperm. (**A**) Number of pups that were obtained by crossing the three tested genotypes of male mice (BRD7^+/+^, n = 16; BRD7^+/−^, n = 17; BRD7^−/−^, n = 11) with normal WT female partners; ***P < 0.0001. (**B**) The serum testosterone, estradiol hormone levels and follicle-stimulating hormone in serum from BRD7^+/+^, BRD7^+/−^ and BRD7^−/−^ male mice were detected using an ELISA kit, and eight mice from each genotype were included in this analysis. (**C**) Gross morphology of the testes and brains from BRD7^+/+^ (left), BRD7^+/−^ (middle) and BRD7^−/−^ (right) mice. (**D**) Comparison of the ratio for testis/body weight among BRD7^+/+^ (n = 12), BRD7^+/−^ (n = 9) and BRD7^−/−^ (n = 14) mice; ***P < 0.001. (**E**) Staining of sperm smear from BRD7^+/+^, BRD7^+/−^ and BRD7^−/−^ mice. The sperms in the slides were stained with eosin. (**F**) The number of epididymal sperms from BRD7^+/+^, BRD7^+/−^ and BRD7^−/−^ mice (***P *<* 0.0001; n = 5). (**G**) Top panel, H&E staining of epididymis from 12-week-old mice. Scale bar =50 μM; Bottom panel, Transmission electron microscopy (TEM) images of the epididymis. The arrows indicate the nuclei of epididymal sperm, and the arrowheads indicate the tails. Scale bar =2 μM.

Our previous study showed high BRD7 expression in the testis. To determine whether BRD7 loss had any impact on male fertility, we mated normal female mice with WT (BRD7^+/+^), Het (BRD7^+/−^) or KO (BRD7^−/−^) male mice. Female mice coupled with WT or Het male mice had an average of 8 pups in each litter, whereas the female mice coupled with homozygous KO males did not produce any pups nor displayed any evidence of pregnancy (Fig. 3A). No differences in the levels of testosterone, estradiol hormone and follicle-stimulating hormone were found among these groups (Fig. 3B). To understand the underlying defect of this observation, we examined the size and morphology of the testes isolated from three cohorts of adult BRD7^+/+^, BRD7^+/−^, and BRD7^−/−^ mice. BRD7^−/−^ mice displayed a smaller testicular size and less weight as well as reduced diameter of seminiferous tubules compared with their BRD7^+/+^and BRD7^+/−^ littermates (p < 0.0001), whereas no difference was found between the BRD7^+/+^and BRD7^+/−^ mice (Fig. 3C,D, Supplementary Fig. S3). However, the proportion of haploid, diploid and tetraploid cells was not affected by the loss of BRD7 (Supplementary Fig. S4). Furthermore, no epididymal sperm was observed in the KO mice (Fig. 3E–G). Together, our results indicate that the loss of BRD7 led to male infertility and the absence of sperm in the epididymis.

### Disruption of BRD7 leads to a complete arrest of spermatogenesis at step 13 of the condensing spermatid

**Figure 4:**
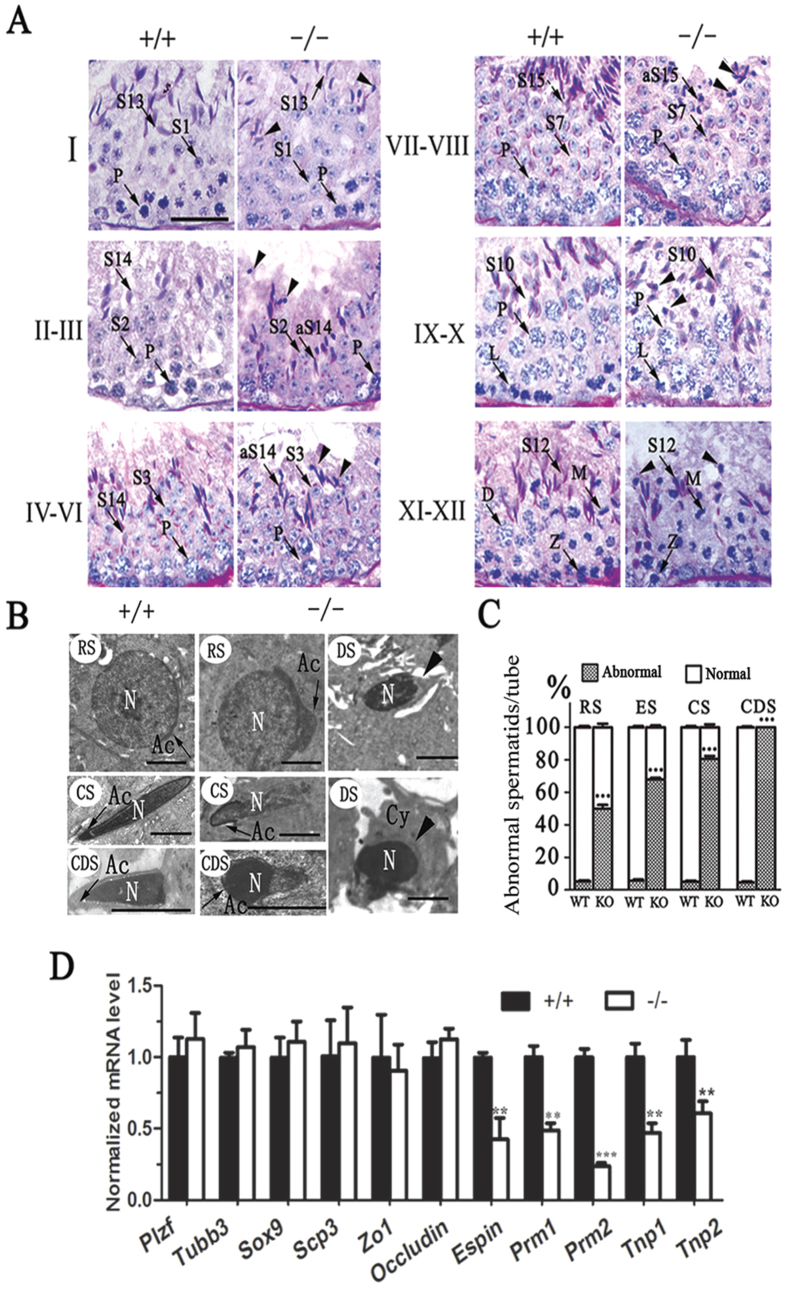
Defective development of elongating spermatids in BRD7^−/−^ mice. (**A**) PAS-H staining of seminiferous epithelia at stages I- XII from BRD7^+/+^ and BRD7^−/−^ mice. Post-meiotic spermatids in steps 1–16 (S1–16) are indicated by arrows. Elongating spermatids begin at step 9 and end at step 16 before being released into the lumen. BRD7^−/−^ mice had abnormal spermatids (aS9-aS16), and the condensing or condensed spermatids were degenerated (arrow heads). Z, zygotene spermatocyte; P, pachytene spermatocyte; D, diplotene spermatocytes; M, meiotic germ cell; Step 1–16 spermatids, S1–16, Scale bar =20 μm. (**B**) TEM image of seminiferous tubules from BRD7^+/+^ and BRD7^−/−^ mice. Scale bar = 2 μm; RS, a deformed acrosome was indicated by an arrow in BRD7^−/−^ mice; CS, abnormalities in nuclear condensation and acrosome formation in BRD7^−/−^ mice; CDS, lack of acrosome and defective head shape in BRD7^−/−^ mice (the arrows indicate the acrosome); DS, degenerative spermatids (indicated by arrowheads), Cy, cytoplasm; RS, round spermatid; CS, condensing spermatid; CDS, condensed spermatid; DS, degenerative spermatid; N, nucleus. (**C**) Statistical analysis of abnormal spermatids in WT and KO mice. Fifty seminiferous tubules for each stage (Stage I-XII) were analyzed, and five adult mice of each genotype were included in the analysis. (**D**) Expression of markers related to different steps of spermatogenesis in BRD7^+/+^and BRD7^−/−^ mice by real-time qPCR (n = 5). “n” indicates the number of mice in each group **P < 0.01; ***P < 0.001.

We further detected the morphology of germ cells in the testis sections during multiple stages of spermatogenesis. Periodic Acid-Schiff (PAS) staining was performed to reveal the dynamic changes in the acrosome formation of round spermatids during successional stages in the seminiferous epithelia of BRD7^+/+^ and BRD7^−/−^ mice. The postmeiotic development of elongating spermatids in the seminiferous tubules of BRD7^−/−^ mice was disrupted and characterized by abnormal morphology in round spermatids (S1–8) and elongating spermatids (S9–11) (Fig. 4A), and massive degeneration was observed in condensing (S12–13) and condensed spermatids (S14–16) (Fig. 4A, arrow heads). The acrosome granules are further highlighted in Supplementary Fig. S5, which shows that BRD7^+/+^ mice presented normal acrosome formation and sickle-shaped heads at step 14 (black arrowheads). However, the BRD7^−/−^ mice showed abnormal acrosomes and developmental morphology (Supplementary Fig. S5, yellow arrowheads). Furthermore, in KO mice, quantitative analysis showed increased proportions of abnormal spermatids (49.95 ± 7.13% of the round spermatids (steps 1–8), 67.84 ± 3.51% of the elongating spermatids (ES, steps 9–11), 80.65 ± 5.8% of the condensing spermatids (CS, step 12–13) and 100% of the condensed spermatids (CDS, steps 14–16)) (Fig. 4C). To support these findings, transmission electronic microscopy (TEM) analysis was further performed to show that these abnormal spermatids are characterized by an irregular head shape in the CS and CDS, an absent or deformed acrosome (Fig. 4B, arrows) and degeneration in BRD7^−/−^ mice (Fig. 4B, DS). The abnormality in round spermatids (RSs) of BRD7^−/−^ mice included a deformed acrosome without asymmetry, whereas the acrosome extended along the nucleus evenly in the wild-type mice (Fig. 4C, RS, arrows). In condensing spermatids and condensed spermatids, an irregular head shape with structural imperfections in acrosome formation was observed (Fig. 4C, CS, CDS). Even condensed spermatids showed globozoospermia-like heads (Fig. 4C, CDS). Moreover, the late spermatids (CS, CDS) in BRD7^−/−^ mice underwent extensive degeneration and were most likely phagocytosed by the cytoplasm of Sertoli cells. We further evaluated the expression of germ cell markers, including Plzf for spermatogonia, Tubb3 and Sox9 for Sertoli cells, SCP3 for spermatocytes, and Prm1, Prm2, Tnp1, Tnp2 and Espin for condensing and condensed spermatids, by real-time qPCR. As expected, the markers for condensing and condensed spermatids in BRD7^−/−^ compared with BRD7^+/+^ mice were markedly down-regulated (Fig. 4E), which is consistent with the reduction in mature spermatids calculated in BRD7^−/−^ mice. Moreover, we further detected the expression of ZO-1 and Occludin for blood-testis barrier evaluation by real-time qPCR, and found no significant difference between BRD7^−/−^ and BRD7^+/+^ mice (Fig. 4E), suggesting that the BRD7 deficiency has no effect on the blood-testis barrier of male mice. These results suggest that BRD7 is required for the late steps of spermatogenesis, particularly for the maturation of elongating spermatids.

### Enhanced apoptosis during germ cell development in BRD7-deficient mice

**Figure 5:**
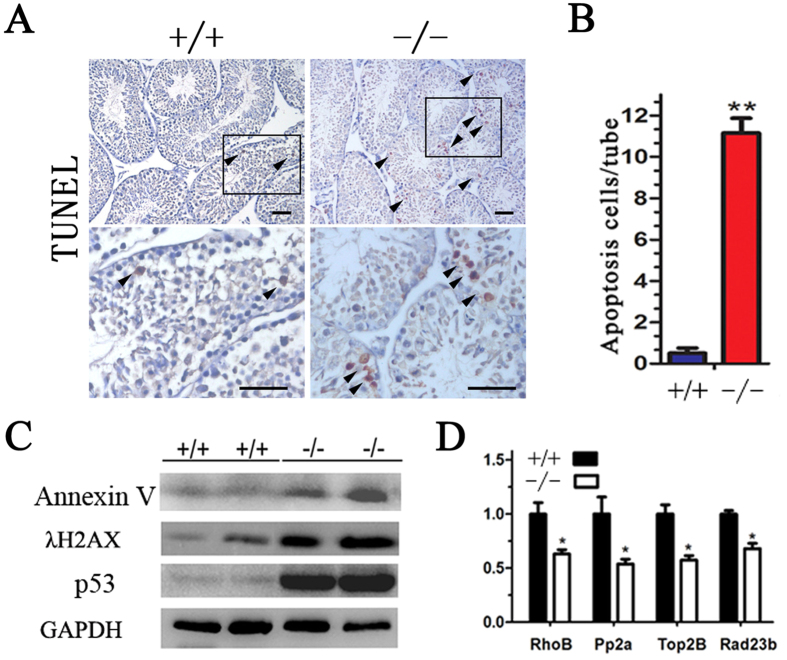
High apoptosis level of germ cells detected in BRD7^−/−^ mice. (**A**) Apoptotic germ cells were detected by TUNEL analysis. TUNEL staining of testis sections from BRD7^+/+^and BRD7^−/−^ male mice. The arrowheads indicate apoptotic germ cells. (**B**) The mean apoptotic cells in each seminiferous tubule. For each mouse genotype analyzed, the numbers of apoptotic cells were counted in 20 random seminiferous tubules (n = 5). Scale bar =50 μm. (**C**) Western blotting analysis of BRD7, Annexin V, P53, γ-H2AX and GAPDH in the testes of BRD7^+/+^ and BRD7^−/−^ mice. (**D**) Real-time qPCR analysis of DNA repair genes in BRD7^+/+^ and BRD7^−/−^ mice (n = 5). “n” indicates the number of mice in each group. *P < 0.05; **P < 0.01.

To determine the apoptosis level in the testes of KO and WT mice, a TUNEL assay was performed. TUNEL-positive germ cells in KO compared to WT mice were markedly increased (Fig. 5A). The number of apoptotic cells per section of epithelium in KO mice was significantly higher than the number in the WT mice (Fig. 5B). The appearance of TUNEL-positive cells was always accompanied by augmented Annexin V expression^20^, which can recognize and clear apoptotic cells^21^. As expected, a high Annexin V level was observed in the BRD7^−/−^ mice testes, as detected by western blotting (Fig. 5C). p53 is a critical mediator of germ cell apoptosis and DNA-damage repair^22^,^23^,^24^. In our study, we found extremely high p53 levels in the testes of BRD7^−/−^ mice but observed extremely low levels in the testes of control mice (Fig. 5C), which suggests that the high apoptosis observed in BRD7^−/−^ testes may be mediated by p53 activation.

To further ascertain the cause of the initiation of germ cell apoptosis, we sought to examine the status of DNA damage and repair in testis sections of BRD7^−/−^ mice. As expected, λH2AX, an endogenous DNA damage indicator, was highly expressed (Fig. 5B), whereas DNA repair genes such as RhoB, Pp2a, Top2B and Rad23B, were transcriptionally down-regulated in the testes of BRD7^−/−^ mice (Fig. 5D). This finding suggests that the high apoptosis observed in the BRD7^−/−^ mice testes may be mediated by the abnormalities in p53 activation and DNA damage repair.

### Association of BRD7 expression with human idiopathic azoospermia with spermatogenesis arrest

**Figure 6:**
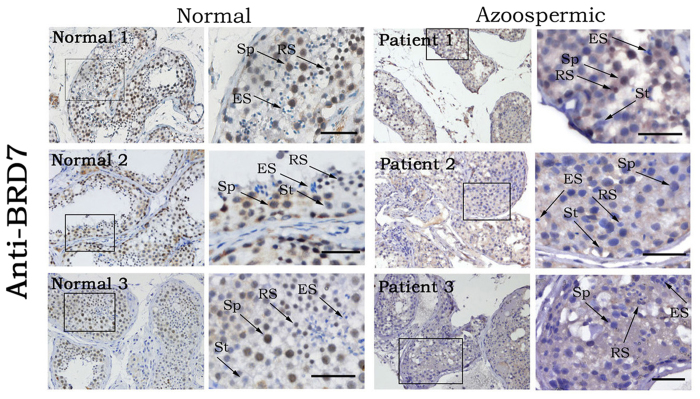
No or low expression of BRD7 was detected in the testes of idiopathic azoospermic patients. The representative images of immunohistochemistry assay for BRD7 expression in testis sections from normal individuals and idiopathic azoospermic patients were shown. Sp, spermatocytes; RS, round spermatids; ES, elongating spermatids; St, Sertoli cells. Scale bar = 50 μm.

BRD7 deficiency can lead to a blockage of spermatogenesis and male infertility in mice, therefore, we associated male infertility resulting from BRD7 disruption with human idiopathic azoospermia. We collected 58 paraffin-embedded testicular biopsies from azoospermia patients and 35 normal biopsies, and all of the patients were pathologically diagnosed with idiopathic azoospermia with spermatogenesis arrest. An immunohistochemistry assay was then performed to detect the expression of BRD7 in the testis sections of azoospermic samples, and normal testicular biopsies were used as controls. As expected, BRD7 is generally expressed in the nuclei of primary spermatocytes and round spermatids whether in normal or azoospermic samples (Fig. 6A), which is similar to the distribution pattern in mice. Among the normal group, all of 35 samples displayed the positive staining of BRD7. However, among the 58 azoospermic samples, BRD7 was only detected in 29 cases, while undetectable in other 29 cases (Table 1), and the intensity of BRD7 staining is totally weaker in BRD7-positive azoospermic samples as compared to the normal samples. These results suggested that BRD7 defect may be associated with the human azoospermia and possibly plays critical roles in human sterility.

## Discussion

BRD7 was initially identified as a novel member of the bromodomain family and contains a functional nuclear localization signal sequence (NLS) and a nuclear export signal sequence (NES)^25^. BRD7 is also a partner of the SWI/SNF complex, a chromatin remodeling complex involved in transcriptional regulation^17^. BRD7 has the capability to bind to specific acetylated histones H3 and H4 through its bromodomain^26^. Many members of the bromodomain family have been reported to bind to acetylated histones and to regulate biological functions involved in obesity, inflammation, carcinogenesis, spermatogenesis, and other diseases^19^,^27^. Several bromodomain proteins are required for spermatogenesis, such as Brdt, a testis-specific double bromodomain BET factor. Brdt contributes to the histone acetylation-mediated programming of the genome by directing genome-wide histone transition and is an important controller of male genome packaging^28^. All of these elements support the hypothesis that BRD7 may be a transcription factor that plays critical roles in the process of normal physiology and disease occurrence.

In this study, we investigated the dynamic expression of BRD7 during testis development and differentiation and spermatid maturation. In the seminiferous epithelium, BRD7 is not expressed in spermatogonia and in the later stages of development, ranging from elongating spermatids to mature sperm. However, BRD7 is highly expressed in the stages from the pachytene to the round spermatid, where spermatogenesis transcription is concluded^28^. Because BRD7 is a critical transcription factor, the loss of BRD7 may affect the transcription of numerous critical genes involved in spermatogenesis. In addition, Sertoli cells also play vital roles in spermatogenesis, which supports the protection and environment of the spermatogenic cells as well as the phagocytosis of apoptotic spermatids and residential bodies in spermiation. From pn 3 to pn 12, BRD7 was present only in Sertoli cells, and its expression in total testis extracts was decreased over time due to the decrease of Sertoli cell in the testis (Fig. 1C,D). From pn 14, BRD7 was associated with the differentiation of pachytene spermatocytes and round spermatids (Fig. 1D). No significant change in BRD7 was observed among pn 23 and pn 45, confirming that BRD7 is not expressed in elongating spermatids, as shown by IHC (Fig. 1C,D). The V-shaped dynamic expression pattern of BRD7 that appeared in postnatal testis development most likely resulted from the proportion of Sertoli cells and multiple spermatid stages of spermatogenesis.

We constructed a global BRD7-knockout mouse model using the EIIα-Cre/*LoxP* and Flp/*FRT* recombination systems, which can mediate site-specific DNA recombination and are being increasingly utilized to study gene function *in vivo*^29^. Using these BRD7-KO mice, we found that the disruption of BRD7 in homozygous (BRD7^−/−^) mice results in complete male infertility and blocking of spermatogenesis, which sheds insights into BRD7’s *in vivo* function. However, male heterozygous (BRD7^+/−^) mice with one mutant allele of BRD7 were viable and fertile, demonstrating that a partial (half) dosage of BRD7 allows complete retention of spermatogenesis and fertility functions. Many genes have been reported to have similar functions in knockout mice^30^,^31^. To confirm that the phenotype of male infertility and abnormal spermatogenesis specifically resulted from BRD7 deficiency in the sperm cells of testes, we further constructed germ-cell-specific BRD7 null mice by crossing BRD7-LoxP mice with Stra-Cre mice. Complete male infertility and defects in sperm cells in the epididymis were observed in these male mice (data not shown), supporting that a BRD7 deficiency in germ cells is the primary cause of male infertility and abnormal spermatogenesis.

Moreover, BRD7 disruption does not affect the sexual response, hormone levels (Fig. 3B) and mating ability of mice; however, the testis was underdeveloped and no mature sperm was observed in the testis epididymis (Fig. 3C,D), which may cause male infertility in KO mice. Furthermore, PAS staining of testis sections of seminiferous tubules showed that KO mice suffered from multiple defects in late spermatid development (Fig. 4). During the round spermatid phase, the acrosome increases in size and begins to spread throughout the anterior nuclear pole^32^. Disrupted acrosome formation is often associated with defective late spermatid development, resulting in deformed head morphology^33^,^34^. In our study, defective acrosome formation in BRD7^−/−^ mice was observed in abnormal spermatids with a globozoospermia-like shape (Fig. 4B), which indicates that abnormalities in acrosome formation influences the formation of spermatid heads in BRD7^−/−^ mice. In the late steps of spermatogenesis, the transition nuclear proteins (TPs) are required for histone displacement, sperm nuclear shaping, chromatin condensation, and maintenance of DNA integrity^35^. The knockout mice with TP1 or TP2 depletion both presented abnormalities in sperm morphology and reduced fertility^36^,^37^. In addition, TP1 mRNA transcription can be activated by the histone demethylase JHDM2A through binding to the core TP1 promoter. Jhdm2a-deficient mice exhibit impaired postmeiotic chromatin condensation and male infertility^38^. Therefore, TP1 and TP2, accompanied with PRM1 and PRM2, are often considered late spermatogenesis markers. In our results, the TP1, TP2, PRM1 and PRM2 mRNA levels were found to be down-regulated in BRD7^/^ mice, which is consistent with reduced amounts of normal late-step spermatids.

Another reason underlying the loss of sperm in the epididymis may be a high apoptosis level in the germ cells in the testes of BRD7^−/−^ mice. Exposure to excess hormones or deprivation of hormones can lead to cellular apoptosis in the testis. The receptors for follicle-stimulating hormone (FSH), estradiol hormone and testosterone are the primary hormonal regulators of spermatogenesis^39^. Our results showed no significant differences in the levels of testosterone, estradiol and FSH between WT and KO mice (Fig. 3), suggesting that BRD7 deficiency-induced germ cell apoptosis is not resulted from the change of these sexual hormones in male mice.

Many apoptotic pathways, including the extrinsic or death receptor pathway and the intrinsic and mitochondrial pathways, influence each other^40^. The p53 pathway is essential for cell growth regulation and apoptosis induced by genotoxic and non-genotoxic stresses^41^. Transgenic mice that constitutively express wild-type p53 in the testis produce few spermatozoa because the majority of the developing spermatids undergo apoptosis^42^. We found a greater number of TUNEL-positive germ cells and a higher expression of the apoptosis marker Annexin V in BRD7^−/−^ mice compared with wild-type mice (Fig. 5A). Moreover, increased accumulation of γ-H2AX and p53 (Fig. 5C) also supply convincing evidence that the germ cells of BRD7^−/−^ mice suffered DNA damage-induced apoptosis with p53 activation. Many mutations in various genes such as Bax and Hspa2 may result in testicular sperm cell apoptosis^43^.

During spermatogenesis, a complex interplay of histone post-translational modifications (PTMs) occur in the nuclei of immature germ cells as they develop into mature spermatozoa^44^. Various chromatin remodeling factors such as SWI/SNF are involved in this process, and dynamic histones acetylation also occurs. BRG1, the catalytic subunit of the mammalian Swi/Snf-like BAF chromatin-remodeling complex, plays an important role in spermatogenesis. Germline-specific Brg1 deletion completely arrests spermatogenesis at the mid-pachytene stage and accumulated DNA damage in germ cells^45^. BRD7 has been identified a component of SWI/SNF complex and most likely serves as critical chromatin remodeling factor during spermatogenesis.

A previous variation analysis of testes from normal and infertile male patients identified a series of genes as biomarkers or targets for the diagnosis and treatment of male infertility such as BRDT, AZF, CREM and PRM2^46^,^47^. Targeting these biomarkers using small molecule inhibitors could be useful as a reversible male contraceptive strategy^48^. To ascertain the clinical value of BRD7, we further investigated the association of BRD7 with male infertility in humans. Interestingly, a similar expression pattern of BRD7 in mouse testis were found in the human data. Moreover, among the azoospermic samples with spermatogenesis arrest, half of them presented negative with BRD7 staining, and even among the BRD7-positive azoospermic samples, BRD7 totally presented lower expression as compared to the normal testis biopsies. Actually, Mathias Wilhelm, *et al.* found that BRD7 is also highly expressed in the adult testis tissues by establishing amass-spectrometry-based draft of the human proteome^49^. Although we demonstrated the association between BRD7 defect and human azoospermic in this study, low or no expression of BRD7 is the cause or result of human azoospermic which need to be further investigated based on series of *in vitro* and *in vivo* experiments. Taken together, our results demonstrate that BRD7 is involved in male infertility and spermatogenesis in mice, and BRD7 defect might be associated with the occurrence and development of human azoospermia.

## Methods

### Animals and ethics statement

Adult C57BL/6 mice were obtained from Shanghai Research Center for Biomodel Organisms (Shanghai, China). All animal experiments in this study conformed to the standards according to the Guide for the Care and Use of Laboratory Animals as published by the US National Institutes of Health (NIH Publication No. 85–23, revised 1996). The procedures of all experiments were approved by the Institutional Animal Care and Use Committee and the Animal Ethics Committee of Central South University.

### Generation of knockout mice

To obtain BRD7-knockout mice that allowed both conditional and global disruption of the BRD7 gene, we used the Cre/loxP and flp/FRT recombination systems to target exons 3 and 4 of BRD7. Exons 3–4 of the BRD7 gene contain 493 nucleotides, and the removal of these two exons results in a frameshift mutation that splices exons 2 and 5. The fragment including exons 2–5 was inserted into the targeting pBR322 vector, in which a single LoxP site before exons 3 and 5, was fused, and an frt-flanked PGK neo cassette was inserted into intron 2 before the LoxP site as a positive selectable marker. The targeting vector was linearized with Not I and electroporated into ES cells under standard conditions. Diagnostic PCR was performed to identify the ES-positive clones with successful homologous recombination using primers spanning from the 5′-arm (15570–19927) or 3′-arm (19721–22483) flanking the PGK-neo cassette, and seven of the 89 ES cells were positive at both sites (Supplementary Fig. S2B). The genomic DNA from two positive clones was digested with Not I, and successful homologous recombination of the BRD7LoxP-neo allele was determined by PCR using the same primers indicated above. One of the positive clones was used to derive male germline BRD7LoxP-neo chimeras. After obtaining the homozygous mice with the BRD7 LoxP-neo phenotype, EIIα-Cre (FVB/N) and FLP (B6J.129S4) mice were used to generated BRD7^−/−^ mice as introduced in the pervious publication^50^.

### Breeding assay and Genotyping of mice

The 12–16 weeks old mice were selected for breeding assays. In the assay, the ratio of male to female was 1:1, and all the wild type female mice mating with male mice (BRD7^+/+^, BRD7^+/−^ and BRD7^−/−^) produced cervical mucus plugs. The breeding success was 100% in the wild-type versus heterozygous mice. If the female mice were not pregnant, up to one month will be maintained together in the course of the assays. The genomic DNA (gDNA) was extracted from tail or ear biopsies using the Mouse Tail DNA Isolation Kit according to the manufacturer’s instructions^51^ and was used as templates in PCR reactions with the primer pairs listed in Table S1 for.

### RT-PCR and real-time qPCR assays

The total testicular RNA from mice over 12 weeks of age was extracted using the TRIzol® Reagent (Invitrogen) and further reversed and transcribed to generate cDNA using the RevertAid First Strand cDNA Synthesis Kit (Fermentas) according to the manufacturer’s protocol. Real-time PCR analysis was then performed to detect the expression of some markers that are specifically expressed in the later steps of spermatogenesis using SBYG® *Premix Ex RTaq*^TM^ II (Tli RNaseH Plus) with the primers listed in Table S2 and using the testicular cDNAs as templates.

### Western blotting analysis

The proteins were then separated by SDS/polyacrylamide gel electrophoresis using a 4.5% stacking gel and a 10% separating gel and transferred to a nitrocellulose membrane (Bio-Rad). After blocking in PBS with 5% non-fat dry milk for 1 h, the membranes were incubated overnight at 4 °C with the primary antibodies in PBS with 5% non-fat dry milk. The following antibodies were utilized: anti-GAPDH and monoclonal antibody (Santa Cruz, 1:1000), anti-mBRD7 polyclonal antibody (ProteinTech, 1:1000), anti-p53 monoclonal antibody (CST, 1:500), anti-ZO-1 antibody (ProteinTech, 1:1000), anti-Annexin V antibody (Biosis 1:200), and anti-gamma H2AX antibody (Millipore, 1:500) were used to detect protein expression.

### Clinical samples

All the experimental protocols were approved by Center for Medical Ethics of Central South University. All of the paraffin-embedded testicular biopsies, which included 58 testicular biopsies from idiopathic azoospermia patients with spermatogenic arrest at spermatid stage and 35 from normal individuals, were obtained from the Second Affiliated Hospital of Hunan University of Traditional Chinese Medicine. The methods were carried out in accordance with the approved guidelines and all the patients provided written informed consent in the study. The patients underwent clinical investigation, including percutaneous epididymal sperm aspiration (PESA) according to WHO guidelines^52^, and were diagnosed as idiopathic azoospermia with spermatogenic arrest at spermatid stage which showed detectable spermatogonias, spermatocytes, spermatids. The azoospermia samples as Sertoli only syndromes or spermatogonia arrest were excluded in the study. Testicular biopsies were performed following the immunohistochemistry instructions provided below.

### Immunohistochemistry (IHC) analysis

Testis sections (μm), fixed with 4% paraformaldehyde, were dehydrated, and after antigen retrieval by microwave irradiation in citrate buffer, the sections were treated with 0.3% H_2_O_2_, PBS and further blocked in 3% BSA/PBS. The sections were incubated with anti-BRD7 antibodies overnight at 4 °C, and a rabbit anti-BRD7 antibody (1:150 dilutions, Santa) was used for the human testicular biopsies and a polyclonal anti-mBRD7 antibody (1:600 dilution, ProteinTech) was used for the mice testes. Labeling was revealed by MaxvisionTM2 HRP-Polymer anti-Rabbit IHC Kit (KIT-5905, FuZhou, China). Sections were counterstained with hematoxylin and analyzed on an Olympus BX51 photomicroscope equipped with an Insight QE Spot camera. The degree of immunostaining of BRD7 in the testis sections was reviewed by two independent observers who were blinded to the clinical data of the patients. For each slide, at least 20 seminiferous tubules were observed and analyzed based on the distribution and intensity of BRD7 staining in testis sections. The undetectable staining for BRD7 was viewed as negative samples, and the detectable staining as positive samples.

### Immunofluorescence of Testes Sections

The preparation of testis slides was processed as previously described in IHC protocol. Antibodies were used as followed: anti-mBRD7 polyclonal antibody (ProteinTech, 1:1000), γH2AX (Millipore 05–636, 1∶1000). Slides were washed three times in PBS, and then incubated for 1 hour at room temperature in 3% NGS, 0.1% Triton-X 100 in PBS containing the relevant secondary antibodies conjugated to Ig-Alexa Fluor 594 or Ig-Alexa Fluor 488 (1∶500 dilutions; Invitrogen). Slides were washed three times in PBS before incubate with DAPI (1∶1000 dilutions; Invitrogen). Images of tissue sections were photographed using a Olympus IX51 fluorescence microscope.

### TUNEL test and Periodic acid-Schiff (PAS-H) and H&E staining assays

The sections were dewaxed and hydrated as described above. The terminal deoxynucleotidyl transferase mediated dUTP-biotin-nick-end-labeling (TUNEL) assay was performed to detect the apoptotic germ cells using the DeadEnd™ Colorimetric TUNEL System (Promega USA) according to the manufacturer’s instructions. For PAS-H staining, the testes were fixed overnight in Bouin’s solution and then transferred to 70% ethanol. Next, 5 μm sections were stained with hematoxylin and Periodic acid-Schiff (PAS) reagent to visualize the acrosome^53^. For H&E staining, the procedure after hydration was performed according to the procedure previously described by Borras^54^. Digital images were acquired with an Olympus BH2 microscope equipped with a Spot RT Slider camera (Diagnostic Instruments, Sterling Heights, MD, USA).

### Sperm counts

Each cauda epididymis was cut with a surgical blade along the side of the corpus epididymis. The excised cauda epididymis was punctured and compressed gently in phosphate-buffered saline (PBS) using the side of a 1-ml disposal syringe needle. The spermatozoa were diluted in PBS, washed three times by centrifugation at 300 g for 5 min and placed into a hemocytometer. The sperm number was counted per sample as previously described^55^.

### Sexual hormone assay

One hundred microliters of whole blood were collected from each 12-week-old male mouse, and eight mice were included in each group (BRD7^+/+^, BRD7^+/−^ and BRD7^−/−^). The serum testosterone, estradiol hormone and follicle-stimulating hormone levels of each group were measured using chemiluminescence immunoassay (CISA) kits (Elabscience Biotechnology Co., Ltd) according to the manufacturer’s instructions.

### Transmission electronic microscopy

Tissues were removed from WT and KO males. The testicular or epididymal tissues were fixed overnight with 2.5% glutaraldehyde (Sigma Aldrich) in 0.1 M phosphate buffer (pH7.4) and subsequently for 2 h in 1.0% osmium tetroxide. The testes were then washed in 0.1 M sodium phosphate buffer and postfixed three times in 1% osmium tetroxide in 0.1 M sodium phosphate buffer at 4 °C. After repeated washing steps, the tissues were dehydrated with a graded series of acetone and embedded in Epon812, DDSA (dodecenylsuccinic anhydride), MNA (methylnadic anhydride), and DMP30 (dimethylaminomethyl phenol) at 60 °C for 24 h. Semi-thin 1 μm-thick sections were routinely stained with toluidine blue for light microscopy. Ultrathin 60–80-nm-thick sections were contrasted with uranyl acetate and lead citrate and examined using a H7700 Hitachi electron microscope. Digital images were captured using a MegaView III digital camera.

### Flow cytometry

Single suspended testicular cells were isolated from 12-week-old BRD7^+/+^and BRD7^−/−^ mice. These cells were incubated with Hoechst 33342 (Sigma)^56^, and flow cytometry was used to analyze the haploid, diploid and tetraploid contents based on the DNA content.

### Measurement of the testis diameter

The diameter of a seminiferous tubule was defined as the shortest distance between two parallel tangent lines of the outer edge of the tubule. Testis sections of 4 mice/group were obtained by optical microscopy using an Olympus IX51 camera x10-micrometer eyepiece coupled with an x10 objective glass (Olympus Japan). The seminiferous tubule diameter was measured using the QCapture Pro software (version 6.0; QImaging).

### Statistical analysis

The comparisons of body weights, organ weights, qPCR data, tubular diameters and hormone levels between the BRD7 KO and control mice were made through Student’s t-test using SPSS 15.0 software. Comparison of the expression of BRD7 in the testis sections from azoospermic samples and control group were made by chi-square. All of the experiments were repeated three times, and a p-value ≤0.05 was considered statistically significant. *P < 0.05; **P < 0.01; ***P < 0.001.

## Additional Information

**How to cite this article**: Wang, H. *et al.* Knockout of BRD7 results in impaired spermatogenesis and male infertility. *Sci. Rep.* **6**, 21776; doi: 10.1038/srep21776 (2016).

## Supplementary Material

Supplementary Information

## Figures and Tables

**Table 1:** BRD7 expression in 35 normal testes and 58 idiopathic azoospermia.

Samples	Numbers	BRD7 staining		P-value
		Negative Positive		
Azoospermia	58	29	29	<0.0001
Normal	35	0	35	
