# Supplementary material for: Knockout of BRD7 results in impaired spermatogenesis and male infertility
Source: Sci Rep. 2016 Feb 16;6:21776. doi: 10.1038/srep21776 (PMC4754950; doi:10.1038/srep21776)
Supplement: Supplementary Information [file srep21776-s1.pdf]

Knockout of BRD7 results in impaired spermatogenesis and male infertility

Heran Wang[1,2,*], Ran Zhao[2,*], Chi Guo[2], Shihe Jiang[2], JingYang[2], Yang Xu[2], Yukun Liu[2], Liqing Fan[3], Wei Xiong[2], Jian Ma[2], Shuping Peng[2], Zhaoyang Zeng[2], Yanhong Zhou[2], Xiayu Li[4], Zheng Li[2], Xiaoling Li[2], David C. Schmitt[5], Ming Tan[5], Guiyuan Li[1,2], Ming Zhou[1,2]

[1]The Affiliated Tumor Hospital of Xiangya Medical School, Central South University, Changsha, Hunan 410013, P.R. China;

[2]Cancer Research Institute, Central South University, Key Laboratory of Carcinogenesis, Ministry of Health, Changsha, Hunan 410078, P.R. China

[3]Institute of reproduction and stem cell engineering, Central South University, Changsha, Hunan 410078, P.R. China

[4]The Third Xiang-Ya Hospital, Central South University, Changsha, Hunan 410013, P.R.China

[5]Mitchell Cancer Institute, University of South Alabama, Mobile, Alabama 36604, USA

*These authors contributed equally to this work.

Correspondence and requests for materials should be addressed to M.Z. (zhouming2001@163.com)

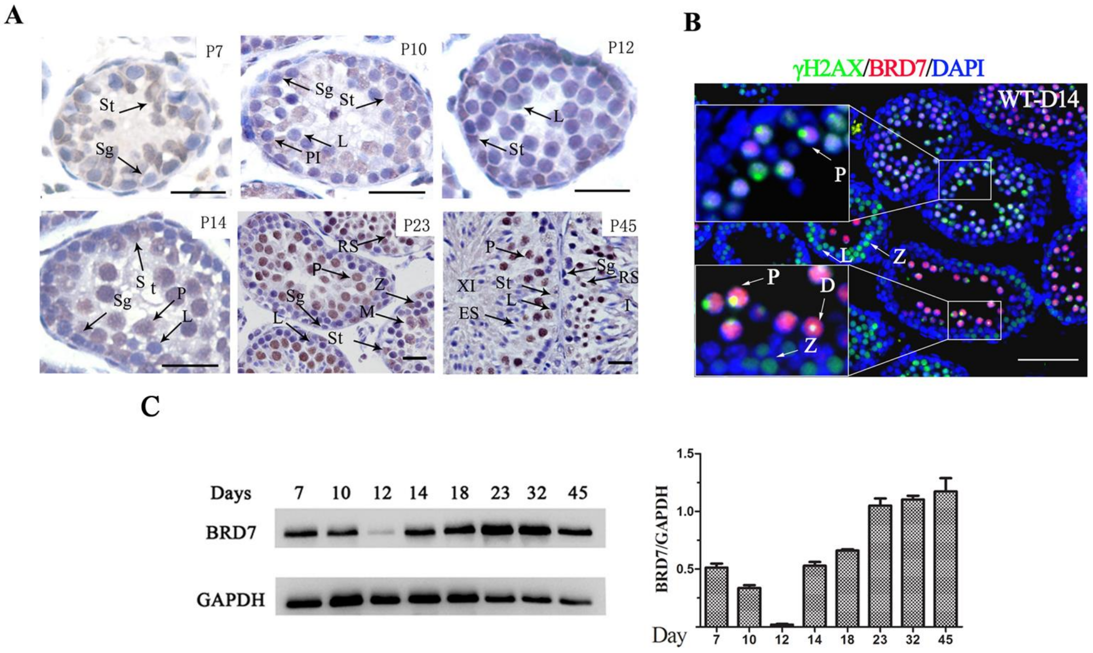

Supplementary Figure S1. Expression pattern of BRD7 in mice testis development. (A) Immunostaining of testicular sections from wild-type mice at different postnatal days (P7, P10, P12, P14, P23 and P45) with anti-BRD7 antibody. The Roman numerals within each tubule denote the stage of the tubule as defined previously. During P7-P45, BRD7 was observed in the nucleus of St (arrows). During P10, BRD7 was undetectable in Sg and PL (arrows). At P12, BRD7 was undetectable in Sg and Z (arrows). At P14, BRD7 was observed in P. During P23, BRD7 was detectable in P, M and RS (arrows). At P45, BRD7 was detectable in the differentiation from P to RS (arrows). St, Sertoli; Sg, spermatogonia; PI, preleptotene spermatocytes; L, leptotene spermatocytes; Z, zygotene spermatocyte; P, pachytene spermatocyte; D, diplotene spermatocytes; M, meiotic germ cell; RS, round spermatid; ES, elongating or elongated spermatids. Scale bar: 50 μm; **(B)** Immunofluorescence of seminiferous tubules from P14 mice showing γ H2AX-positive pachytene or diplotene spermatocytes (Green, XY body) express BRD7 (red); Leptotene and Zygotene spermatocytes expressing γH2AX (Green, area of the whole nuclei) don't express BRD7 (red)**.** Scale bar: 100μm. **(C)** Left panel, western blots of BRD7 in the testis from postnatal P7, P10, P12, P23, and P45. Right panel, histogram of gray-scale scanning analysis of the western blots.

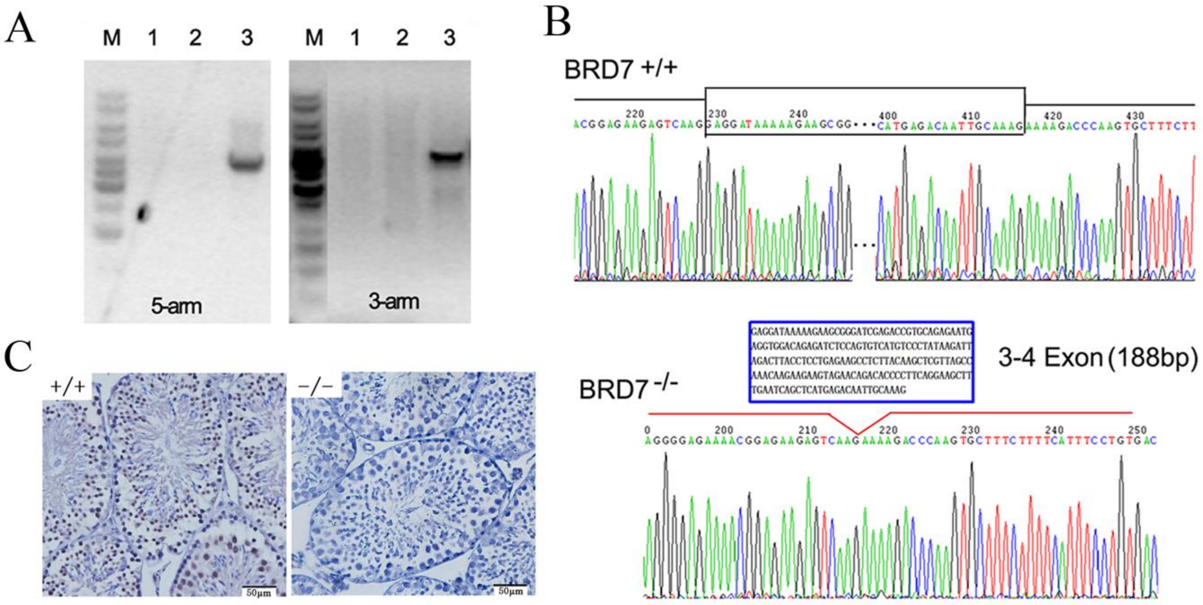

Supplementary Figure S2. Generation and identification of BRD7-knockout mice. (A) RT-PCR Identification of heterozygous mice with both 5'-arm and 3'-arm generated from chimeric male mice. The numbers represent the different mice. (B) Sequence identification according to exons 3 and 4 from $BRD7^{+/+}$ and $BRD7^{-/-}$ mice. (C) IHC analysis of testis sections from $BRD7^{+/+}$ and $BRD7^{-/-}$ mice with BRD7 antibody. Scale bar = 50 μm.

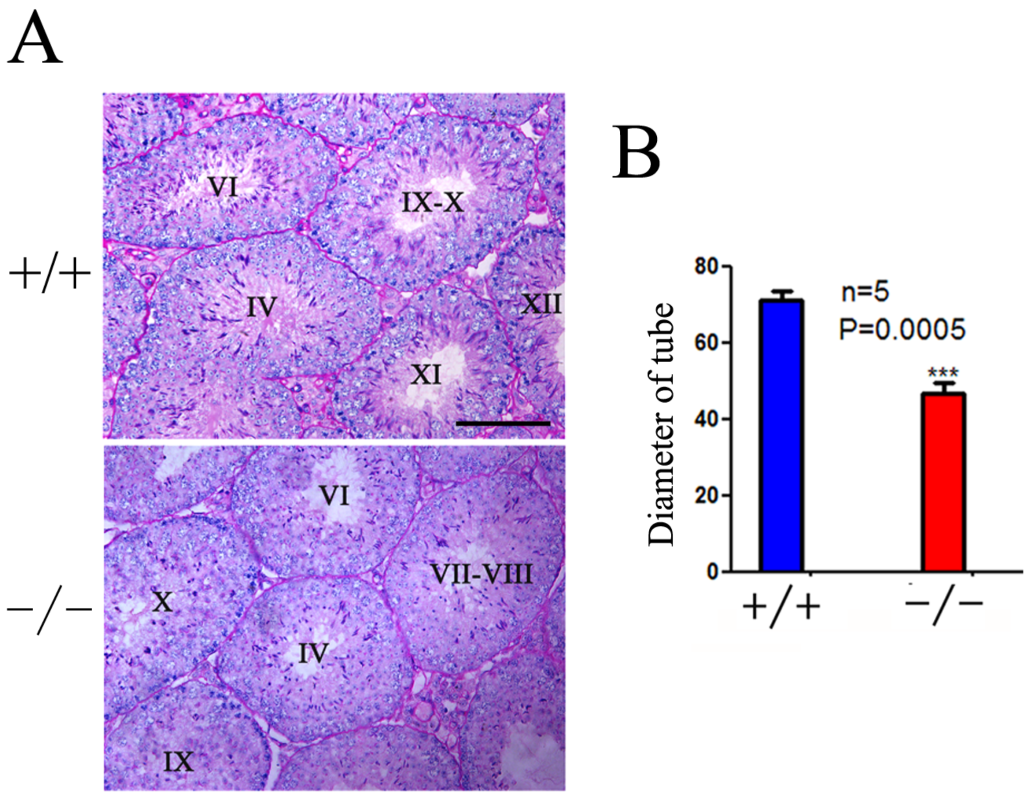

Supplementary Figure S3. Comparison of the seminiferous tubules and testicular cells between BRD7$^{+/+}$ and BRD7$^{-/-}$ mice. (A) PAS-H staining of testis sections from BRD7$^{+/+}$ and BRD7$^{-/-}$ mice. Roman number indicates different stage of eipthelium. Scale bar = 100 μm. (B) Comparison of the seminiferous tubule diameter of the testes from BRD7$^{+/+}$ and BRD7$^{-/-}$ mice. One hundred tubules were analyzed for each mouse.

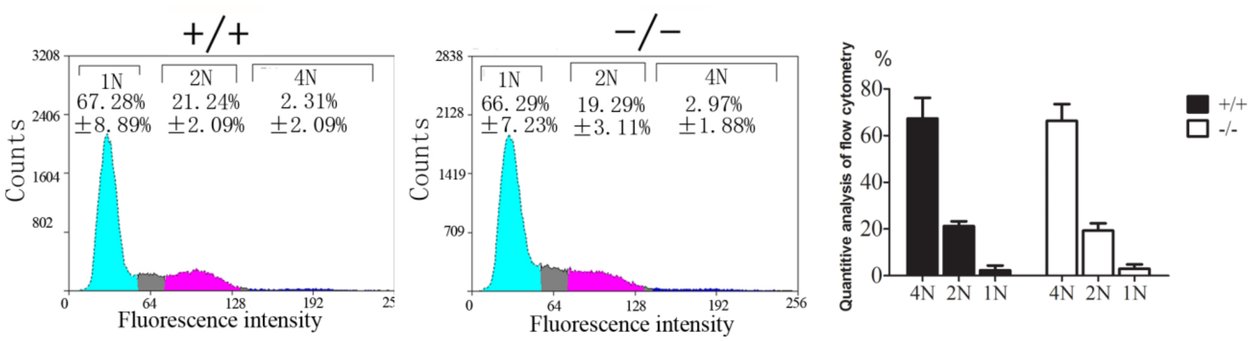

Supplementary Figure S4. Flow cytometry analysis of germ cell types (tetraploid, diploid and haploid). Flow cytometric analysis from DNA histograms of representative isolated testicular cells from $BRD7^{+/+}$ and $BRD7^{-/-}$ mice. Right panel show the histogram of flow cytometric analysis. 3 mice were used in each group.

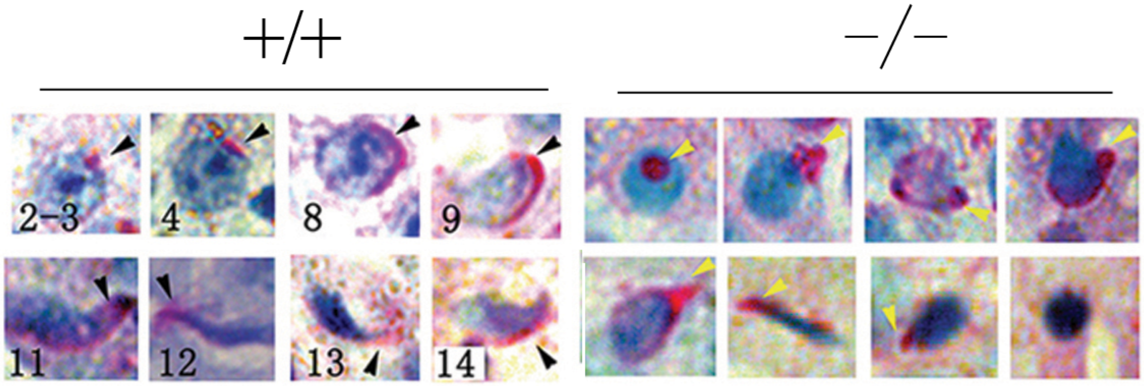

Supplementary Figure S5. PAS-H staining of spermatids at different steps. Numers indicate the step of spermatids. Black arrows show the acrosome of wild type during development. Yellow arrows the abnormal acrosome of BRD7$^{-/-}$ acrosome.

Supplementary Table S1. Primers used for the identification of mouse genotypes by PCR

Sort	Name	Forward primers	Reversed primers	Product	Aims
1	Frt-neo	GGCTATTCGGCTATG ACTGG	ATACTTTCTCGGCAG GAGCA	288 bp	Neo-cassette
2	Cre	GCGGTCTGGCAGTA AAAACTATC	GTGAAACAGCATTG CTGTCACTT	100 bp	EⅡα-Cre
3	LoxP1	AGTTCCTATTCTCTA GAAAG	TCCCGCTTCTTTTTAT CCTC	215 bp	LoxP1 site
4	LoxP2	CAAACCACACCTCTG CCTTAG	CACCCTCTTGGGACT GCTT	241 bp	LoxP2 site
5	LoxP3	AGTTCCTATTCTCTA GAAAG	ACCCTCTTGGGACTG CTTTT	149 bp	LoxP3 site
6	WT	TCCAGTAGATGGCA GCAAAA	ATTCTCTGCACGGTC TCGAT	190 bp	WT allele

Supplementary Table S2. Primers used for the detection of marker genes specifically expressed in some steps of spermatogenesis by real-time PCR

Sort	Gene	Forward primers	Reversed primers
1	Anxa5	TATCCCCCACCACATCATCT	TATCCCCCACCACATCATCT
2	Espin	CCACAGGCTACCTCTCTTGC	CTGGTACAGCAGCCACTTCA
3	Plzf	CCATGATCAAGCACCTGAGA	GTGGCCCTTCATGTGTTTCT
4	Sox9	CTGAAGGGCTACGACTGGAC	TACTGGTCTGCCAGCTTCCT
5	Tubb3	GGCATGGATGAGATGGAGTT	CCGATTCCTCGTCATCATCT
6	Scp3	AAGCATTCTGGGAAATCTGG	GGAGCCTTTTCATCAGCAAC
7	Occludin	CGGTACAGCAGCAATGGTAA	CTCCCCACCTGTCGTGTAGT
8	Gapdh	GGGTGTGAACCACGAGAAAT	ACAGTCTTCTGGGTGGCAGT
9	Zo1	ACTCCCACTTCCCCAAAAAC	ACAGCTTTAGGCATGGTGCT
10	Tnp1	GCATGAGGAGAGGCAAGAAC	GGATCGGTAATTGCGACTTG
11	Tnp2	TCGACACTCACCTGCAAGAC	CTTGTATCTTCGCCCTGAGC
12	Prm1	CAAAATTCCACCTGCTCACA	CATCTGCTCCTGCTTTTGCT
13	Prm2	CCACAAGAGGCGTCGGTCAT	CCTTGGCTCCAGGCAGAATG
14	RhoB	ACTATGTGGCGGACATCGAG	CACTTCTCGGGGATGTTCTC
15	Top2B	GAAAGCGTGGAAAGAAGCAC	CGCCCTTTCGTATCTCTCTG
16	Rad23b	CGCAGCTTCCACATTAGTGA	GGAATTCCCGTGAGCAAGTA
17	Pp2a	TCGATCGCCTACAGGAAGTT	AGGCCATTGGCATGATTAAA
